# Identification of HLA class I-restricted immunogenic neoantigens in triple negative breast cancer

**DOI:** 10.3389/fimmu.2022.985886

**Published:** 2022-11-02

**Authors:** Belén Aparicio, David Repáraz, Marta Ruiz, Diana Llopiz, Leyre Silva, Enric Vercher, Patrick Theunissen, Ibon Tamayo, Cristian Smerdou, Ana Igea, Marta Santisteban, Cristina Gónzalez-Deza, Juan J. Lasarte, Sandra Hervás-Stubbs, Pablo Sarobe

**Affiliations:** ^1^ Centro de Investigación Médica Aplicada (CIMA), Universidad de Navarra, Pamplona, Spain; ^2^ Centro de Investigación Biomédica en Red de Enfermedades Hepáticas y Digestivas (CIBEREHD), Pamplona, Spain; ^3^ IdiSNA, Instituto de Investigación Sanitaria de Navarra, Pamplona, Spain; ^4^ Oncología Médica, Clínica Universidad de Navarra, Pamplona, Spain

**Keywords:** triple negative breast cancer, neoantigens, immunogenicity, polyepitopic vaccines, HLA-A*02:01 epitopes

## Abstract

Immune checkpoint inhibitor (ICI)-based immunotherapy in triple negative breast cancer (TNBC) is achieving limited therapeutic results, requiring the development of more potent strategies. Combination of ICI with vaccination strategies would enhance antitumor immunity and response rates to ICI in patients having poorly infiltrated tumors. In heavily mutated tumors, neoantigens (neoAgs) resulting from tumor mutations have induced potent responses when used as vaccines. Thus, our aim was the identification of immunogenic neoAgs suitable as vaccines in TNBC patients. By using whole exome sequencing, RNAseq and HLA binding algorithms of tumor samples from a cohort of eight TNBC patients, we identified a median of 60 mutations/patient, which originated a putative median number of 98 HLA class I-restricted neoAgs. Considering a group of 27 predicted neoAgs presented by HLA-A*02:01 allele in two patients, peptide binding to HLA was experimentally confirmed in 63% of them, whereas 55% were immunogenic *in vivo* in HLA-A*02:01^+^ transgenic mice, inducing T-cells against the mutated but not the wild-type peptide sequence. Vaccination with peptide pools or DNA plasmids expressing these neoAgs induced polyepitopic T-cell responses, which recognized neoAg-expressing tumor cells. These results suggest that TNBC tumors harbor neoAgs potentially useful in therapeutic vaccines, opening the way for new combined immunotherapies.

## Introduction

Triple negative is the most aggressive breast cancer subtype. In the metastatic scenario, median overall survival is about 15 months, as compared to other biological subtypes that exceed 4 years (1). Nowadays, improvement in survival has been shown dramatically in the HER2 overexpressed subtype due to the incorporation of new targeted therapies, missing this benefit in advanced triple negative breast cancer (TNBC) (2). Thus, new therapeutic strategies are mandatory in TNBC patients. Pembrolizumab, an anti PD-1 monoclonal antibody, has been approved by FDA in the neoadjuvant scenario in stage II and III TNBC patients, due to the improvement shown in pathological complete responses as well as in event-free survival (EFS), when combined with anthracyclines and taxanes plus platinum salts-based chemotherapy (3).

Regarding immunotherapy, TNBC has several features that suggest its suitability to therapies based on immune checkpoint inhibitors (ICI). When compared with other types of breast tumors, TNBC has an enriched lymphocytic infiltrate, higher levels of tumor mutational burden (TMB) and overexpression of immune checkpoints (4). Despite these general features, TNBC tumors have a heterogeneous microenvironment, with differences in the amount and types of immune infiltrating cells (5, 6). ICI, like anti-PD-1/PD-L1 antibodies, in combination with chemotherapy, have been approved for positive PD-L1 metastatic TNBC (3, 7). However, as in other tumors, a proportion of patients do not respond, making necessary the development of alternative strategies with improved efficacy. In this regard, immunotherapies should be tailored according to features of the tumor immune microenvironment. As opposed to those patients with highly infiltrated tumors that contain lymphocytes expressing the immune targets, there are other patients with tumors with lower presence of immune cell populations and downregulation of most immune targets (8). Vaccination has been traditionally used to promote antitumor immunity; however, despite successful activation of tumor-specific T-cells, the immunosuppressive tumor microenvironment, including ligands for immune checkpoints, may hinder effector functions of these cells (9). Therefore, combination of immunostimulatory vaccines with ICI is an attractive approach to enhance response rates to immunotherapy in patients with cold tumors. Several vaccines have been used in preclinical models and TNBC patients, using as antigens folate receptor, alpha-lactalbumin, MUC-1, Brachiury or cancer-testis antigens, among others (10–12). Our group recently reported results on a cellular vaccine based on tumor lysate-pulsed dendritic cells (13). These vaccines were usually safe: thus clinical and immune responses were detected in many cases, however no impact on survival was shown. A strategy to improve vaccine potency has been the selection of more immunogenic antigens (9). Many antigens used in vaccination protocols are self-antigens whose repertoire of T cells has been negatively selected during thymic development, resulting in a subset of T cells with lower affinity. Moreover, autologous tumor cell lysates, despite their content of personalized antigens, may potentially include an overrepresentation of self-antigens that would mask the repertoire of targetable antigens. Neoantigens (neoAgs), antigens arising as a consequence of tumor mutations, are new molecules non subjected to clonal deletion, and therefore, may induce potent tumor-specific responses (14). The amount of neoAgs in a tumor associates with its TMB, which is positively correlated with response to ICI (15). Immune responses against these antigens have been detected in patients responding to ICI, suggesting their relevance as rejection antigens (16, 17). Accordingly, they have gained great interest as candidates to be included in vaccines (18–21). Due to these reasons, we aimed to demonstrate that TNBC tumors could harbor mutations potentially considered as neoAgs, and that these sequences are immunogenic and could be included in vaccines.

## Patients and methods

### Patients and samples

The studies involving human participants were reviewed and approved by Ethical and Scientific Committees, Universidad de Navarra (ref # 2017.210). The patients/participants provided their written informed consent to participate in this study. Eight patients with II-III stage TNBC diagnosis (only one patient with IV oligometastatic stage) were included in the study. Median age was 51 (35–74) years old. All patients received neoadjuvant chemotherapy based on sequential anthracyclines and taxanes ± platinum salts plus radiation therapy. Seven patients were evaluable for response and pathological complete response (residual cancer burden, RCB=0) was reached in 71.4% of the patients (2 patients with RCB=2). No patients received adjuvant systemic therapy.

A needle tumor biopsy was extracted at diagnosis and preserved at -80°C in RNAlater. Mononuclear cells were isolated by Ficoll-gradient from EDTA-anticoagulated whole blood extracted at diagnosis and preserved at -80°C as dried pellet. Tumor breast biopsies and blood samples and data from patients were provided by the Biobank of the University of Navarra and were processed following standard operating procedures approved by the Ethical and Scientific Committees.

### Whole exome sequencing and RNAseq

For Whole exome sequencing (WES), genomic DNA was purified from tumor biopsy and blood samples (normal tissue) (Maxwell 16 Tissue DNA Purification Kit, Promega) and preserved at -80°C. WES library (SureSelect Human All Exon V6) and sequencing (NovaSeq6000 150PE 18Gb 100X on target) was subcontracted to Macrogen Inc (Korea). WES data were analyzed with an in-house bioinformatics pipeline. Briefly, alignments of WES to the reference human genome build hg38 was performed using BWA-mem algorithm (0.7.17). Duplicates were marked using Picard’s MarkDuplicates tool. Insertion and deletion (indel) realignment and base recalibration were performed according to Genome Analysis Toolkit (GATK) best practices. SAMtools was used to create tumor and normal pileup files. Different mutation callers were used to call somatic non-synonymous SNVs (single nucleotide variants) [Mutect3.8, Varscan2, SomaticSniper (version 1.0.5.0), and Strelka(version 2.9.2)]. Mutations were annotated using Annovar. Only mutations annotated as exonic and non synonymous_(SNV mutation) in RefGene were considered. To generate an initial list of putative mutations for evaluation, the following filters were used: a tumor and normal coverage of greater than 6, a variant allele frequency (VAF) ≥ 5%, variant read counts ≥ 4. WES data were used for HLA genotyping using HLA-HD algorithm (22).

RNA was purified from tumor biopsy (Maxwell 16 LEV simplyRNA Tissue Kit, Promega) and preserved at -80 °C. RNA sequencing (RNAseq) was subcontracted to Macrogen Inc (Korea). Briefly, complementary DNA libraries were constructed from High quality RNA (1 μg) using TruSeq stranded Total RNA Sample Prep Kit. Library was subjected to paired-end sequencing using the NovaSeq platform (10 Gb 100PE 100Mreads/sample). Alignments were performed using the STAR method to human genome build hg38. Duplicates were marked and Sorted using Picard’s MarkDuplicates tool. Reads were then split and trimmed using GATK SplitNTrim tool. In/Del realignment and base recalibration were performed using GATK toolbox. Gene counts were calculated using featureCount (23), Cufflinks (24) and HTseq (25).

### TMB calculation and neoantigen selection

For TMB calculation, SNVs detected by Mutect2 (3.8) were used. For neoantigen selection, SNVs called by 2 or more callers were considered. TMB was calculated as the ratio between the number of mutations and the size of the sequenced regions (35.7 Mb). For those patients with available RNAseq, quartiles of FPKM were calculated. Mutations identified by WES analysis were considered as “expressed mutations” if at least one of the gene counter algorithms was expressed in 3^rd^ or 4^th^ quartile. Once mutations were filtered, 29-mer amino acid sequences, containing the mutated residue at position 15, were designed. These sequences were applied to NetMHCPan 4.0 to predict peptide binding to patients’ own HLA class I molecules.

### Cell lines

T2 cells (26), used in HLA binding experiments, and MC703 cells (a fibrosarcoma cell line generated in HHD mice; a kind gift of Dr. Matthias Leisegang; Berlin, Germany), used for *in vitro* T cell assays, were grown in RPMI 1640 medium (Gibco) containing 10% FBS (Sigma) and antibiotics (100U/mL penicillin, 100 μg/mL streptomycin, Gibco). Platinum-A (Plat-A) (Cell Biolabs, Inc.) were cultured in Plat-A medium (DMEM-Glutamax, Gibco), FCS 10%, sodium pyruvate 1%, essential amino acids 1%, HEPES (all from Gibco), and antibiotics, supplemented with Puromycin (1μg/ml) and Blasticidin (10 μg/ml).

### Binding assays to HLA-A*02:01 molecules

T2 cells (2.5 × 10^5^/well) were incubated overnight at 37°C in 96-well microplates with peptides at 100 μM in RPMI 1640 medium containing 10% FBS and antibiotics (complete RPMI). They were washed and stained with FITC-labeled anti-HLA-A*02 (Genetex) (2 mg/mL, 15 min at room temperature) and mean fluorescence intensity (MFI) was determined by flow cytometry using a FACSCantoII (Becton Dickinson) flow cytometer and FlowJo software (Tree Star Inc.). Peptide binding was expressed as Fluorescence index (FI) using the following formula: (MFI with peptide – MFI without peptide)/MFI without peptide.

### Peptides

Nine- and fifteen-mer peptides containing HLA-A*02:01 epitopes were purchased from GeneCust (Boynes, France) with >90% purity. They were solubilized in PBS containing 10% DMSO and preserved at -20°C.

### Plasmids

Plasmids pBK-T-SFV-b12A-TMGP69 and pBK-T-SFV-b12A-TMGP73 used in immunization experiments have a cytomegalovirus (CMV) promoter driving expression of the Semliki Forest Virus (SFV) vector, which contains the SFV replicase, and a subgenomic promoter driving the expression of the inserts of interest. Each insert was fused to the b1 SFV translation enhancer using the 2A self-protease from foot and mouth disease virus as a linker. The inserts contain a signal peptide from MHC class I, followed by selected 15-mer neoAgs located in tandem and the MHC class I trafficking domain (MITD) sequence (27). Inserts encoding the neoAgs (TMGP69 and TMGP73) were obtained from Genscript and cloned into MSCV-IRES-Thy1.1 DEST and pBK-T-SFV-b12A (28) using BglII&SalI and Apa I sites, respectively. MSCV-IRES-Thy1.1 DEST was a gift from Anjana Rao (Addgene plasmid # 17442). Plasmids pMSCV-TMGP69-IRES-Thy1.1, pMSCV-TMGP73-IRES-Thy1.1 encoding the neoAgs and control plasmid (MSCV-IRES-Thy1.1 DEST) were used to prepare retrovirus for transduction experiments.

### Retrovirus production

Retroviral particles were generated using Plat-A cell-mediated transfection. Plat-A cells (8×10^5^ cells/well) were seeded 24h before transfection in 6-well plates in 2 mL/well of infection medium (Plat-A medium without antibiotics). 20-24h later, once the cells had reached 70% confluence, 500μL/well of a mix containing plasmids and Lipofectamine 2000 (Thermo Fisher Scientific) were added. The mixtures contained 3 μg/well of transgene carrying plasmid, 2 μg/well of pMD2.G (helper plasmid) and 10 μL/well of Lipofectamine 2000, and were prepared in OPTIMEM medium according to the manufacturer´s protocol. pMD2.G was a gift from Didier Trono (Addgene plasmid # 12259). Plat-A medium was changed 24h post-transfection. The supernatant containing the retroviruses was collected 48h and 72h post-transfection. Debris was removed by centrifugation at 2000rpm for 1 minute. Supernatants were kept at 4°C until cell transduction.

### Cell transfection and selection

MC703 cells were transduced twice (two consecutive days) with retrovirus-containing supernatants in the presence of polybrene (10 μg/mL) (Sigma). Efficiency of transfection (90%) was checked at day 4 from infection by measuring the surface expression of CD90.1 (Thy1.1) protein by staining with phycoerythrin-labeled anti-mouse CD90.1 (Thy1.1) (clone OX-7) mAb by flow cytometry. Transduced cells were used to evaluate the T cell response as described below.

### Mice and immunization

HHD-DR1 mice encoding human HLA-A*02:01 and HLA-DRB1*01 (29) were obtained from Dr. F. Lemonnier (Paris, France) and bred in our facilities in pathogen-free conditions. The animal study was reviewed and approved by Ethics Committee for Animal Research (Universidad de Navarra; ref# 045-19). When using peptides, they were immunized subcutaneously with 100 nmoles/mouse combined with polyI:C (Amersham) and anti-CD40 (FGK4.5; Bioxcell), both being administered at 50 μg/mouse. For immunization with pBK-T-SFV-b12A-TMGP69 and pBK-T-SFV-b12A-TMGP73 plasmids, mice were anesthetized with ketamine (Richter Pharma, 0.66 mg/kg) and rompun (Bayer, 8 mg/kg) and injected with 10 μg of plasmid intradermally in the base of the tail. They next received an electroporation using an ECM 830 square wave electroporation system (BTX), by placing a needle array electrode at the injection site injection immediately after immunization. Electroporation consisted in two pulses of 1,125 V/cm for 50 μs followed by 8 pulses of 275 V/cm for 10 ms. They were boosted at two weeks. In all cases, seven days after the last immunization, mice were sacrificed, and spleens were obtained.

### Evaluation of murine T cell responses

Most T cell responses were evaluated by using an IFN-γ ELISPOT Set from BD-Biosciences as described (30). In brief, spleens were removed, homogenized, erythrocytes were lysed and cells (8×10^5^/well) were stimulated with different peptide concentrations (10-0.01 μM). To analyze recognition of neoantigen-presenting tumor cells, irradiated (200 Gy) MC703 cells (8×10^4^ cells/well), transduced with retroviruses encoding neoantigens or with control vector, were cocultured with splenocytes (8×10^5^/well) obtained from immunized mice. In all cases, 24 h later ELISPOT plates were developed and spot-forming cells were counted with an ImmunoSpot automated counter using Immunospot Image Acquisition 4.5 and Immunospot 3 software.

In some cases, T cell activation was determined by flow cytometry. Splenocytes were stimulated for 4 hours with peptides (10 μM) in the presence of GolgiStop and GolgiPlug (BD-Biosciences) and antiCD107a-FITC (BD-Pharmingen). Next, they were stained with anti-CD3ϵ-Percp-Cy5, CD4-FITC and CD8-BV421 (BioLegend). Then, cells were fixed and permeabilized with BD Cytofix/Cytoperm™ Fixation/Permeablization Kit and intracellularly stained with IFNγ-PE and TNFα-BV510 antibodies (BioLegend). Dead cells were excluded from the analysis using Maleimide (PromoKine). Samples were acquired with a Cytoflex cytometer (Beckman Coulter) and data analyzed using FlowJo software.

### Analysis of human T cell responses

*In vitro* priming of T cell responses and evaluation of peptide immunogenicity was carried out as described. Briefly, monocytes were purified from PBMC obtained from HLA-A*02:01 healthy donors by using CD14 microbeads (Miltenyi) and cultured for one day in complete RPMI medium containing rhGM-CSF (Miltenyi; 10 ng/ml) and rhIL-4 (Inmunotools; 2 ng/ml) for their differentiation into dendritic cells (DC). Next, rhTNF (Miltenyi; 10 ng/ml), IL-1β (Immunotools; 10 ng/ml) and PGE2 (Sigma-Aldrich; 1 µM) were added for DC maturation. One day later, cells were collected, loaded with peptide (10 µg/mL) for 1 hour and washed 3 times. DC were co-cultured (ratio 1:30) with CD8 T cells purified from the CD14^-^ fraction using CD8 microbeads (Miltenyi), in complete medium containing anti-human CD28 mAb (Biolegend; 0.5 µg/mL). Cells were fed on days 3, 7, 10, 14 and 17 with culture medium containing IL-2 (Proleukin, 20U/ml).

NeoAg-specific responses were evaluated at day 21 of co-culture by using a human IFN-γ ELISPOT set (BD-Biosciences) following manufacturer’s instructions. Expanded CD8 T cells (5 x 10^4^/well) were stimulated with peptides (10 µg/mL) and DC (10^4^/well) for 24 h. Next, wells were washed, incubated with conjugate antibody and developed. Spot forming cells were counted as described above.

### Statistical analysis

Statistical analyses (Student’s t test and one-way ANOVA with Bonferroni’s multiple comparison test) were performed with GraphPad Prism (GraphPad) software version 7. P<0.05 was taken to represent statistical significance.

## Results

### Tumor mutational burden and neoAg burden in TNBC patients

In order to define neoAgs in TNBC tumors we carried out WES and RNAseq studies to identify mutations that could originate non-synonymous SNVs (nsSNVs) and generate new peptide sequences with a different antigenicity profile. WES studies allowed us to identify a median of 60 mutations (range 19-103) that corresponded to a median TMB of 1.68 Muts/Mb (**Table 1** and **Supplementary Table S1**). For those patients with material available for RNAseq we analyzed gene expression and identified those mutated genes with demonstrated expression. This narrowed the number to a median of 35 mutations (range 31-69) (**Supplementary Table S2**).

**Table 1:** Mutational and neoAg load in TNBC patients.

Patient ID	# mutations*	TMB (Muts/Mb)	NeoAgs Class I
CAM-69	48	1.34	106
CAM-73	103	2.89	155
CAM-75	60	1.68	35
CAM-76	78	2.18	65
CAM-82	19	0.53	38
CAM-83	79	2.21	158
CAM-85	60	1.68	141
CAM-88	47	1.32	90
Mean	61.75	1.73	98.50
Median	60	1.68	98.00

(*) non-synonymous single nucleotide variants.

HLA class I molecules expressed by patients were identified using sequencing data (**Supplementary Table S3**) and all potential peptides containing mutated residues were selected for prediction of peptide binding to HLA. By using a threshold of 500 nM in affinity and a % Rank <2%, a median of 98 potential neoAgs were predicted (**Table 1**, **Supplementary Table S4**).

Although our main interest in this work was on HLA class I-restricted neoAgs, in order to have a general neoAg profile we also predicted putative neoAgs with binding capacity to HLA class II molecules. Considering an affinity threshold of 250 nM and a % Rank < 10%, predictions yielded a median of 146 HLA class II-restricted neoAgs (**Supplementary Table S5**).

### HLA binding ability and immunogenicity of HLA-A*02:01-predicted neoAg peptides

**Figure 1:**
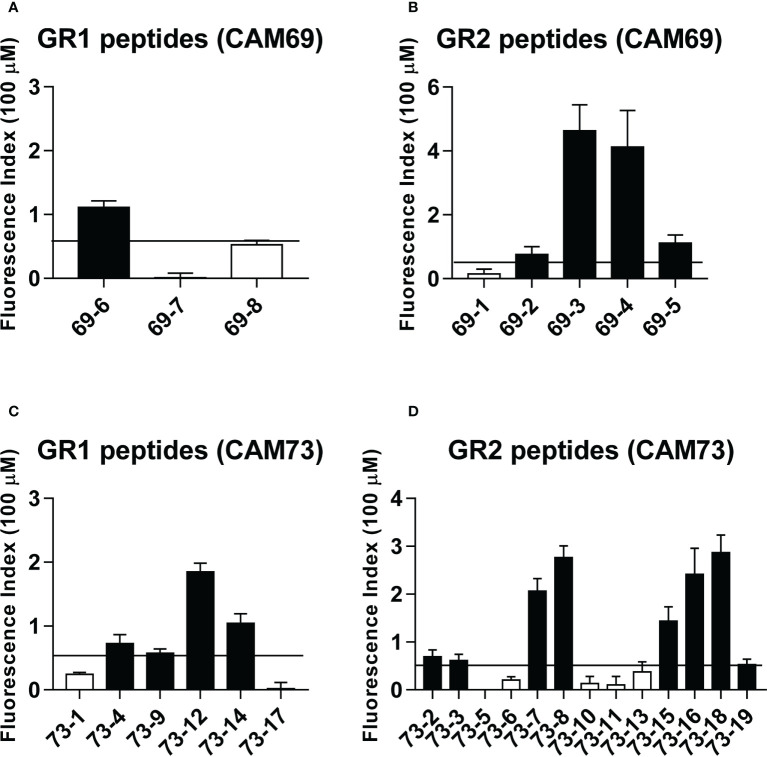
Binding of predicted peptide epitopes to HLA-A*02:01 molecules. Peptides from patients CAM69 **(A, B)** and CAM73 **(C, D)** belonging to GR1 and GR2 groups were tested at 100 μM in binding assays using T2 cells. Results are expressed as Fluorescence Index (FI) and correspond to the mean of duplicate samples in two experiments. Positive binding was considered when FI > 0.5 (horizontal line) and binder peptides are shown with black bars.

After *in silico* prediction of putative neoAgs, we analyzed those presented by HLA class I molecules, to test their immunogenicity and demonstrate their potential applicability in vaccination strategies. As a proof of concept, and due to the availability of appropriate experimental tools, we focused on HLA-A*02:01-restricted peptides, choosing patients CAM69 and CAM73 as representative (**Tables 2**, **3**). Moreover, to reduce the number of neoAgs to be tested, we narrowed the selection to those 9-mer peptide neoAgs potentially presented by this HLA allele with a binding affinity < 250 nM and % Rank < 2, since most 10- and 11-mer peptides contained these 9-mer peptides. Although all mutated (MUT) peptides had a putative HLA binding capacity, we distinguished a first group (GR1) with predicted binding for the MUT but not for the WT version and a second group (GR2) with predicted binding for both the MUT and WT versions. After discarding those peptides that were difficult to synthesize with the amount and purity required, 8 and 19 peptides for patients CAM69 and CAM73, respectively, were selected. One third of the peptides belonged to GR1. *In vitro* binding assays to HLA-A*02:01 demonstrated that 62 and 63% of MUT peptides selected in patients CAM69 and CAM73, respectively, bound to this allele (**Figure 1**).

**Table 2:** HLA-A*02:01-restricted neoAgs in patient CAM69.

				Mut			WT		
Group	Peptide	Gene Name	Mut Aa	Sequence	Aff (nM)	% Rank	Aff (nM)	% Rank	DAI*
GR1	CAM69-6	DNAH8	S2680L	A**L**IPTLLSL	19.5	0.26	2036.5	5.88	104.4
	CAM69-7	PAX2	V224F	HL**F**WTLRDV	87.6	0.97	336.3	2.27	3.8
	CAM69-8	CATSPER1	L527F	VLDF**F**LMQT	171.6	1.50	283.7	2.05	1.7
	CAM69-ns2	CFAP69	S738F	GL**F**AEDFVT	127.2	1.24	2435.9	6.45	19.2
	CAM69-ns3	MUC3A	I833L	G**L**SGSLPMM	96.7	1.04	39787.1	70.10	411.4
GR2	CAM69-1	KLHL29	W663R	SLLDN**R**NLV	54.9	0.68	8.9	0.10	0.2
	CAM69-2	LYG2	R203Q	FVNDIIA**Q**A	33.5	0.45	50.2	0.63	1.5
	CAM69-3	TTK	N630I	**I**MLEAVHTI	4.1	0.02	8.4	0.09	2.0
	CAM69-4	CATSPER1	L527F	AMAVLDF**F**L	7.7	0.08	11.2	0.13	1.5
	CAM69-5	CATSPER1	L527F	**F**LMQTHSFA	8.3	0.09	88.8	0.98	10.7
	CAM69-ns1	RAD9A	R146C	HML**C**APARV	48.1	0.61	251.3	1.91	5.2

(*) DAI: Differential aggretopicity index: Affinity of WT sequence/affinity of mutated sequence.

Bold residues correspond to mutated amino acids.

**Table 3:** HLA-A*02:01-restricted neoAgs in patient CAM73.

				Mut			WT		
Group	Peptide	Gene Name	Mut Aa	Sequence	Aff (nM)	% Rank	Aff (nM)	% Rank	DAI*
GR1	CAM73-1	PDIA6	G309A	VLPHILDT**A**	138	1.30	13530.7	17.91	98.0
	CAM73-4	CTNNB1	P505R	**R**LIKATVGL	29.5	0.40	1802	5.51	61.1
	CAM73-9	ARHGEF28	G187A	SQFFLCLP**A**	107.3	1.10	4771.4	9.22	44.5
	CAM73-12	PLXNA4	D597Y	**Y**LSEMDGLV	7.8	0.08	6425.4	10.93	823.8
	CAM73-14	LYZL2	M50V	FSLGNWIC**V**	151.9	1.39	1782.5	5.48	11.7
	CAM73-17	SLC2A3	V16F	LIFAIT**F**AT	144.8	1.35	573.4	3.03	4.0
GR2	CAM73-2	SLC4A10	I775T	VL**T**DYAIGI	15.3	0.19	5.3	0.04	0.3
	CAM73-3	SLC4A10	I775T	CMVL**T**DYAI	237.7	1.84	222.7	1.76	0.9
	CAM73-5	OR5H15	P275T	NMVE**T**LFYT	11.5	0.14	18.9	0.25	1.6
	CAM73-6	OR5H15	P275T	MVE**T**LFYTV	115.5	1.16	71.4	0.84	0.6
	CAM73-7	CLRN1	F155L	VMIL**L**ASEV	46.3	0.59	17.2	0.22	0.4
	CAM73-8	CLRN1	F155L	IL**L**ASEVKI	67.3	0.80	44.4	0.57	0.7
	CAM73-10	CDC23	S304F	NMDTF**F**NLL	126.4	1.23	148.3	1.37	1.2
	CAM73-11	SLC36A2	M102V	FIACHC**V**HI	21.4	0.29	20.3	0.28	0.9
	CAM73-13	WHRN	Q325H	KVGD**H**ILEV	20.2	0.28	47.4	0.60	2.3
	CAM73-15	LYZL2	M50V	SLGNWIC**V**A	65.5	0.79	30.4	0.41	0.5
	CAM73-16	SLC2A3	V16F	ALIFAIT**F**A	29.8	0.40	164.1	1.46	5.5
	CAM73-18	ACAD10	Q144H	VMTEAIT**H**I	35.7	0.47	46.4	0.59	1.3
	CAM73-19	SETD4	Q181P	SL**P**PLFAEA	42.2	0.55	52.1	0.65	1.2

(*) DAI: Differential aggretopicity index: Affinity of mutated sequence/affinity of WT sequence.

Bold residues correspond to mutated amino acids.

**Figure 2:**
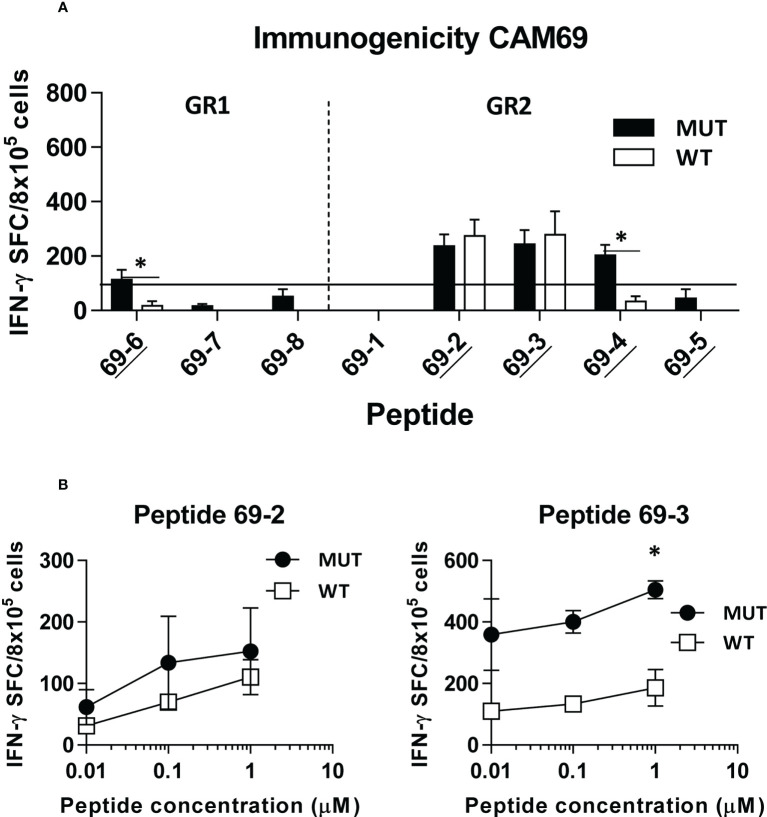
*In vivo* immunogenicity of predicted peptide epitopes from patient CAM69. HHD-DR1 mice (n=4/group) were immunized with peptide pools (4 peptides/pool; 100 nanomoles of each peptide/pool) plus poly(I:C) and antiCD40 adjuvants at days 0 and 7. One week after the boost they were sacrificed and splenocytes were stimulated with individual peptides (either mutated or WT version at 10 μM) **(A)** and response evaluated by using an IFN-gamma ELISPOT assay. Responses were considered positive when SFC > 50. Results correspond to the sum of 2-3 independent experiments. Peptides experimentally proven as binders are underlined. **(B)** Lymphocytes showing positive recognition of the WT version were titrated with lower peptide concentrations. (*P<0.05; MUT vs WT peptide).

**Figure 3:**
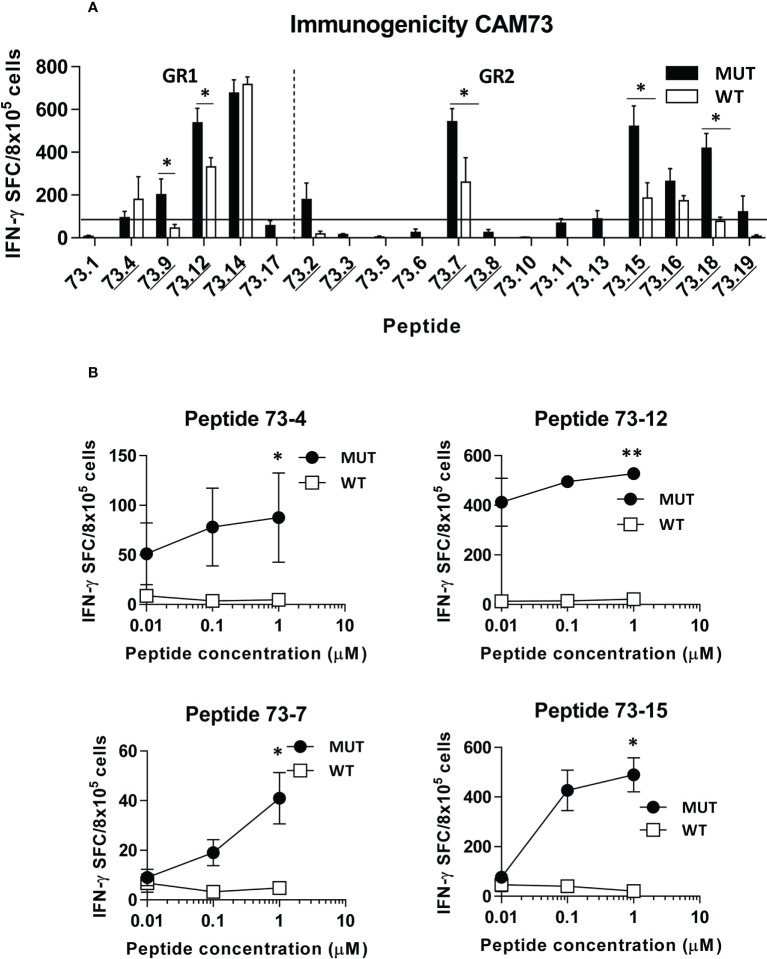
*In vivo* immunogenicity of predicted peptide epitopes from patient CAM73. HHD-DR1 mice (n=4/group) were immunized with peptide pools (4-5 peptides/pool; 100 nanomoles of each peptide/pool) plus poly(I:C) and antiCD40 adjuvants at days 0 and 7. One week after the boost they were sacrificed and splenocytes were stimulated with individual peptides (either mutated or WT version at 10 μM) **(A)** and response evaluated by using an IFN-gamma ELISPOT assay. Responses were considered positive when SFC > 50. Results correspond to the sum of 2-3 independent experiments. Peptides experimentally proven as binders are underlined. **(B)** Lymphocytes showing positive recognition of the WT version were titrated with lower peptide concentrations. (*P<0.05; **P<0.01; MUT vs WT peptide).

**Figure 4:**
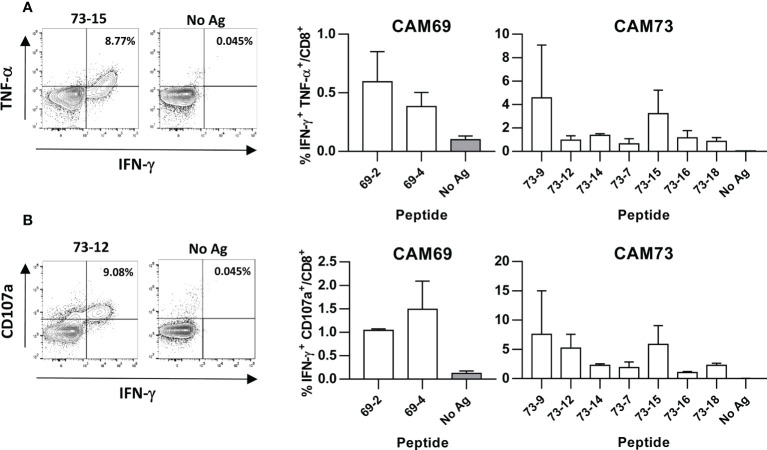
Induction of polyfunctional T cell responses by neoantigen vaccines. HHD-DR1 mice (n=3-4/group) were immunized with peptides (100 nanomoles) plus poly(I:C) and antiCD40 adjuvants at days 0 and 7. One week after the boost, they were sacrificed and splenocytes were stimulated with or without peptides (10 μM) and response evaluated by flow cytometry measuring the percentage of CD8 T cells expressing IFN-γ and TNF-α **(A)** and IFN-γ and CD107a **(B)**. Representative examples of peptide 73-12 and 73-15 (left panels) and summarized results of all peptides (right panels) are shown.

Immunogenicity experiments carried out in HHD-DR1 mice, transgenic for HLA-A*02:01, showed that 50% and 63% of peptides from patients CAM69 and CAM73, respectively, were immunogenic. For patient CAM69 all peptides with proven experimental binding capacity (binder peptides as determined in **Figure 1**), except peptide 69-5, were immunogenic (**Figure 2A**). None of non-binder peptides elicited an immune response. For patient CAM73, 10 out of 12 binder peptides induced an immune response (**Figure 3A**). At the same time, we checked recognition of WT peptides by T cells induced by neoAgs. We observed that peptides WT69-6 and WT69-4 were not recognized when using the initial screening concentration (10 μM) (**Figure 2A**). Regarding peptide WT69-3, despite recognition at this high concentration, it did not elicit IFN-γ secretion at lower concentrations (**Figure 2B**). Similar studies with patient CAM73 demonstrated that, except peptide 73-14, remaining immunogenic peptides induced T cells that, either at the screening concentration or after titration experiments, preferentially recognized the MUT but not the WT version (**Figures 3A, B**). We also demonstrated that, according to the selection criteria, WT versions of immunogenic GR1 peptides did not bind to HLA-A*02:01 molecules, whereas most GR2 peptides did bind (**Supplementary Figure S1**). Finally, we observed that these immunogenic peptides induced polyfunctional T cell responses, inducing not only IFN-γ, as found in ELISPOT assays, but also TNF-α (**Figure 4A**) or the cytotoxicity marker CD107a (**Figure 4B**). These results indicate that these neoAgs induce T cell responses with the capacity to discriminate between MUT and WT sequences, despite the binding capacity of GR2 WT peptides, supporting their use as tumor-specific vaccines.

### Immunization with neoAg-containing polyepitopic vaccines induces tumor-specific T cell responses

**Figure 5:**
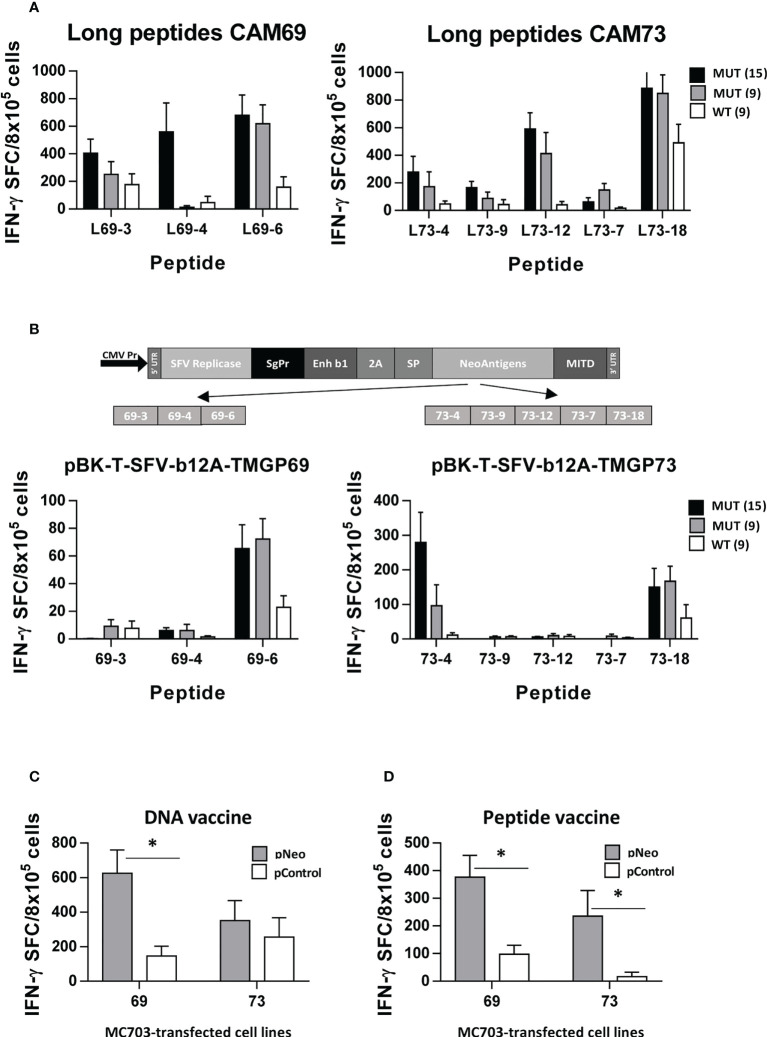
Polyepitopic vaccines induce responses recognizing neoantigen-expressing cells. HHD-DR1 mice (n=4/group) were immunized at days 0 and 7 with 15-mer peptide pools (3-5 peptides/pool) plus poly(I:C) and antiCD40 adjuvants **(A)** or at days 0 and 14 with a plasmid encoding the SFV replicase plus the 15-mer neoAg epitopes from patients CAM69 or CAM73 designed in tandem **(B)**. One week after the last immunization they were sacrificed and splenocytes were stimulated with MUT (15- and 9-mer) peptides or with the WT 9-mer peptides and responses were evaluated by ELISPOT. **(C)** Splenocytes from mice shown in B were stimulated with MC703 tumor cells transfected with a plasmid encoding the neoAgs used in the vaccine or the control Thy1.1 gene and responses were measured as above. **(D)** Splenocytes from HHD-DR1 mice (n=4/group) immunized with peptides CAM69-3, CAM69-4 and CAM69-6, or peptides CAM73-4, CAM73-9, CAM73-12, CAM73-7 and CAM73-18, were stimulated as in C and responses were determined by ELISPOT. (*P<0.05; pNeo vs pControl).

After identification of immunogenic neoAgs, we designed polyepitopic vaccines containing the most immunogenic epitopes, and tested them using two vaccination strategies. For patient CAM69, peptides 69-3, 69-4 and 69-6 were selected, whereas peptides 73-4, 73-7, 73-9, 73-12 and 73-18 were chosen for patient CAM73. These vaccines included either peptide pools adjuvanted with poly(I:C) and antiCD40 or pBK-T-SFV-b12A-TMGP69 and pBK-T-SFV-b12A-TMGP73 DNA vectors, encoding the Semliki Forest virus replicase followed by the neoAgs in tandem, to generate autoreplicative RNAs that would increase antigen expression. To mimic natural antigen processing occurring in the original antigens, these vaccines included elongated versions (15-mers) of each neoAg. In general, peptide vaccines were more immunogenic than the DNA constructs (**Figures 5A, B**). In the case of patient CAM69, both vaccines induced responses against peptide 69-6, which specifically recognized the MUT but not the WT version. Regarding the other two neoAgs, they were immunogenic only as peptides, recognizing better the 15-mer than the 9-mer mutated peptide. For patient CAM73, responses against neoAgs 73-4 and 73-18 were induced by both vaccines. NeoAgs 73-7 and 73-9, and more clearly 73-12, were only immunogenic when used as peptides. In all cases, lymphocytes showed specific recognition of the MUT vs the WT version of immunogenic neoAgs.

Finally, to demonstrate recognition of processed antigens in the context of tumor cells, we prepared transfectants of MC703 cells expressing the neoAgs used for vaccination. We demonstrated that T cells induced by DNA vaccines, mainly for those corresponding to patient CAM69, specifically recognized tumor cells expressing the neoAgs but not control cells (**Figure 5C**). Since responses induced by the DNA construct encoding neoAgs derived from patient CAM73 were of lower magnitude than those induced by the corresponding peptide vaccines, we repeated these experiments but using peptide-induced T cells. As shown in **Figure 5D**, these T cells specifically recognized neoAg-expressing tumor cells.

### Neoantigen peptides induce human T cell responses

**Figure 6:**
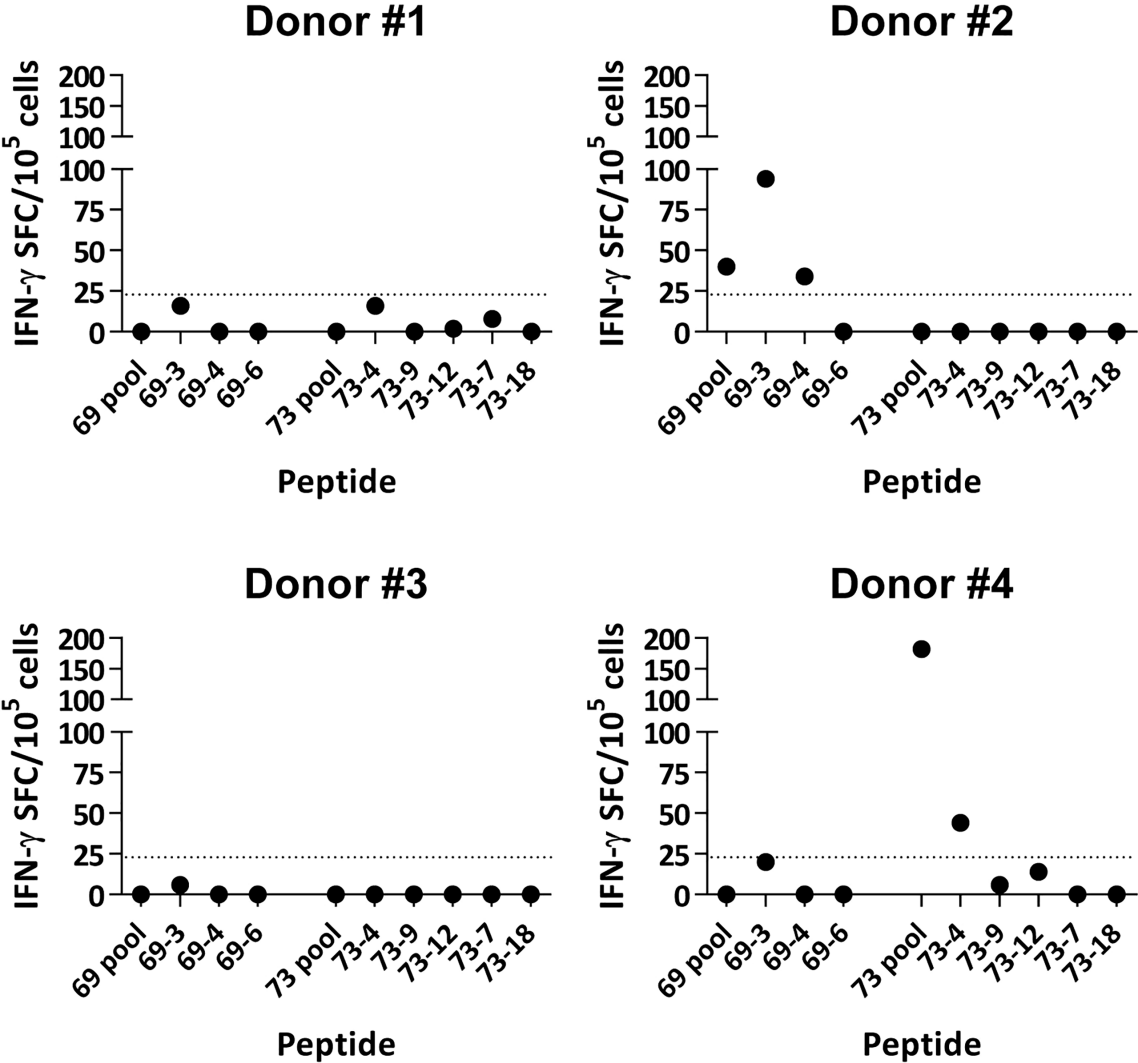
*In vitro* immunogenicity of TNBC neoAgs with human T cells. CD8 T cells obtained from four healthy HLA-A*02:01^+^ donors were stimulated *in vitro* with peptide-pulsed dendritic cells and expanded with IL-2. Three weeks later, T cells were harvested and peptide recognition was measured in ELISPOT assays using peptides and autologous DC. Results show recognition of peptides pools and individual peptides. Values obtained in negative control wells without antigen have been subtracted. The dotted line corresponds to the 20 SFC threshold defining positive responses.

After identification of HLA-A*02:01-restricted immunogenic neoAgs in the murine system, we checked their immunogenicity in humans. The lack of samples belonging to patients initially used for neoAg identification prompted us to use the peptides in *in vitro* priming assays using T cells from four HLA-A*02:01^+^ healthy donors. Peptide recognition assays by T cells obtained after peptide priming and expansion with cytokines showed that two individuals had responses against some of these neoAg peptides (**Figure 6**). More precisely, donor #2 recognized peptides 69-3 and 69-4, and donor #4 recognized peptide 73-4. These results demonstrate that immunogenic neoAgs identified by using the murine *in vivo* vaccination studies are also immunogenic in the human setting.

## Discussion

Vaccines can induce tumor-specific T cells that would generate an enriched lymphocytic infiltrate, increasing thus the possibility of responding to ICI. Infiltrating T cells have been found in TNBC patients (4). However, heterogeneous results have been observed in terms of T cell infiltration, suggesting a different potential benefit after ICI therapies. It has been proposed that neoAg-specific T cells play a prominent role in ICI efficacy, since TMB (as a surrogate marker of potential neoAgs) correlates with the response rate to these therapies (15). Although TNBC has been described as a tumor with a mutational load higher than other breast tumors (4), it is well known that, similar to tumor infiltrates, there is a wide spectrum of mutational burden between patients (31). Thus, in order to develop personalized vaccines in TNBC patients to increase T cell responses, in a cohort of eight patients we have analyzed the mutational load, the potential HLA class I-restricted neoAgs derived from these mutations and finally, we tested their immunogenicity in a humanized murine model. WES results obtained in our cohort, considering only non-synonymous SNVs, yielded a median of 62 mutations/patient, which corresponds to a TMB value of 1.7 Muts/Mb, in the range of those reported in previous works (4, 32, 33), which positions TNBC in the category of tumors with intermediate TMB. Analyses of expression of mutated sequences revealed that about 65% of mutations were located at expressed genes. A high variability regarding expression of mutated genes with potential neoAg-coding capacity has been reported in TNBC, ranging from 35-50% in some cases (4, 34), to more than 80% in others (35). The sample size and the filtering criteria used to define expression in the different studies may account for these discrepancies, as well as for the differences with our results. By using these mutations, we predicted HLA class I-restricted potential neoAgs encoded by mutated genes, observing a median of 98 neoAgs. In general, the number of predicted neoAgs was higher with respect the number of mutations, presumably due to the prediction of peptides with different length sharing the same binding core, the presence of different binding cores containing the same mutation or even to neoAgs potentially presented by different HLA alleles in the same individual.

Different criteria have been used to select predicted peptides. In our case, in addition to a binding threshold for all mutated peptides, we distinguished two groups regarding the binding capacity of the WT peptide. In the first group (GR1), where WT peptides were poor or null binders, an important proportion of peptides contained the mutation at HLA-anchoring residues (e.g. peptides CAM69-6, CAM73-9). However, in the second group (GR2) all peptides contained the mutation at non-anchor residues. In this group, some mutations increased HLA binding (peptides CAM73-4, CAM73-12), whereas others did not increase HLA binding, but presumably generated a different contact site with the TCR (peptides CAM69-4, CAM73-7, CAM73-15 and CAM73-18). In both groups, we have been able to identify immunogenic peptides with capacity to induce T cells discriminating between MUT and WT peptides, indicating that not only the binding capacity, but also the position of the mutation may help to identify neoAgs. Indeed, it has been recently shown the relevance of the position of the mutated amino acid in neoAg selection (36, 37).

Recent studies have analyzed the presence of neoAgs in TNBC tumors (4, 35) and in some cases, their immunogenicity has been demonstrated by using T cells from patients (34, 38, 39). In all cases, and in agreement with our results, the number of neoAgs and the proportion of them with confirmed immunogenicity indicates that the mutational load found in TNBC patients would be sufficient to generate a neoAg-based vaccine. Indeed, in our case, in the two patients whose potential neoAgs have been tested for immunogenicity, positive results have been obtained. Moreover, in addition to considering a single HLA class I allele, our studies are focused on SNVs, without including INDELs and other mutations, which have also shown to encode neoAgs (40). Indeed, for these two patients, 55% of initially predicted neoAgs for HLA-A*02:01 turned out to be immunogenic. Nevertheless, we have demonstrated that some neoAgs identified in murine vaccination experiments were immunogenic *in vitro* using human cells from healthy donors. We have recently reported equivalent results for immunogenicity of neoAgs identified in hepatocellular carcinoma patients (41), demonstrating not only the activity of HLA class I-restricted neoAgs but also for class II binding peptides. Thus, our current results obtained in TNBC patients suggest that the TMB found may originate a sufficient number of neoAgs for vaccination. In fact, it has been recently reported the results of a phase I clinical trial of a neoAg-based DNA vaccine in 18 TNBC patients, where authors vaccinated with an average of 11 neoAgs per patient (42), confirming the feasibility of this approach.

As mentioned above, neoAg-based vaccines could be a promising strategy to prime immunity against target antigens relevant in ICI-based therapies. Indeed, among the group of combined immunotherapies for TNBC that include vaccines and ICI [reviewed in (43)], a few of them are based on neoAgs (NCT03199040, NCT03606967 and NCT03289962). Different approaches are being tested in these protocols, according to the type of ICI (anti-PD-L1 with or without anti-CTLA-4), the combination with chemotherapy (gemcitabine, nab-paclitaxel) or the type of vaccine (DNA vaccine, RNA vaccine or long peptides). In our case, we have evaluated the immunogenicity of two vaccination modalities, including peptides and a DNA vaccine encoding an autoreplicative RNA. Although both were immunogenic, stronger results were obtained with peptides, a strategy successfully used in neoAg vaccines in other tumors (20, 21). We do not know the reasons behind these differences, whether they are specific for the particular neoAgs used in this work or whether it is a consequence of the strong adjuvants (poly(I:C) and antiCD40 mAb) used in combination with peptide vaccines. Immunodominance or competition between epitopes after processing may restrict the repertoire of responses when using a single polypeptide construct as opposed to the use of individual peptides, as we demonstrated (44). Since strong T cell responses have been obtained in neoAg-based clinical trials in patients using peptides or RNA, further experiments are required to elucidate the relative potency of our vaccination strategies.

In summary, our sequencing and immunogenicity studies carried out in a cohort of TNBC patients demonstrate that these patients harbor a sufficient number of immunogenic neoAgs suitable for vaccine development, setting the basis for future combinatorial therapies containing vaccines and ICI.

## Data availability statement

The datasets presented in this study can be found in online repositories. The names of the repository/repositories and accession number(s) can be found below: GEO (refs PRJNA851929 and GSE206998).

## Ethics statement

The studies involving human participants were reviewed and approved by Comité Ético de Investigación Clínica (Departamento de Salud, Navarra). The patients/participants provided their written informed consent to participate in this study. The animal study was reviewed and approved by Comité de Ética de Experimentación Animal, Universidad de Navarra.

## Author contributions

BA, MS, CS, JL, SH-S and PS conceived and designed experiments. MS, CG-D, patient recruitment. BA, DR, MR, DL, LS, EV, PT, IT, CS and AI performed the *in silico*, *in vitro* and *in vivo* studies. BA, SH-S, JL and PS analyzed data and wrote the manuscript. SH-S and PS supervised the project. MS, SH-S, JL and PS, funding. All authors reviewed and approved the manuscript.

## Funding

This work was supported by grants from Gobierno de Navarra (DIANA project, ref 0011-1411-2017-000029 and Socrates project, ref 0011-1411-2022-000088) to MS, JJL, SH-S and PS, from Instituto de Salud Carlos III (project PI20/00260) co-funded by the European Union (ERDF, “A way to make Europe”), and the “Murchante contra el cáncer” initiative to PS, Instituto de Salud Carlos III (project PI20/00415) co-funded by ERDF “A way to make Europe” to CS. SH-S receives funding from ISCIII/FEDER, UE (PI18/00556), and Gobierno de Navarra (Departamento de Salud (045–2017)) co-financed (50%) with FEDER funds (UE, FEDER 2014-2020 “Una manera de hacer Europa”). JJL is also funded by Ministerio de Ciencia e Innovación (PID2019-108989RB-I00, PLEC2021-008094 MCIN/AEI/10.13039/501100011033).

## Acknowledgments

We particularly acknowledge the patients for their participation and the Biobank of the University of Navarra for its collaboration. Authors thank Virginia Villar for her help with sample management. We also thank Thomas Blankenstein’s lab for providing MC703 cells.

## Conflict of interest

MS has received honoraria from Daiichi Sankyo, Pfizer and Gilead and travel support from Gilead.

The remaining authors declare that the research was conducted in the absence of any commercial or financial relationships that could be construed as a potential conflict of interest.

## Publisher’s note

All claims expressed in this article are solely those of the authors and do not necessarily represent those of their affiliated organizations, or those of the publisher, the editors and the reviewers. Any product that may be evaluated in this article, or claim that may be made by its manufacturer, is not guaranteed or endorsed by the publisher.

## Supplementary material

Supplementary Table 1 List of identified mutations.

Supplementary Table 2 Mutations with confirmed expression by RNAseq.

Supplementary Table 3 HLA class I alleles expressed by patients.

Supplementary Table 4 Prediction of binding to HLA class I molecules of mutated and wild type peptides.

Supplementary Table 5 Prediction of binding to HLA class II molecules of mutated and wild type peptides.
