# Supplementary material for: Identification of HLA class I-restricted immunogenic neoantigens in triple negative breast cancer
Source: Front Immunol. 2022 Nov 2;13:985886. doi: 10.3389/fimmu.2022.985886 (PMC9666480; doi:10.3389/fimmu.2022.985886)
Supplement: Supplementary file 6 [file DataSheet_1.pdf]

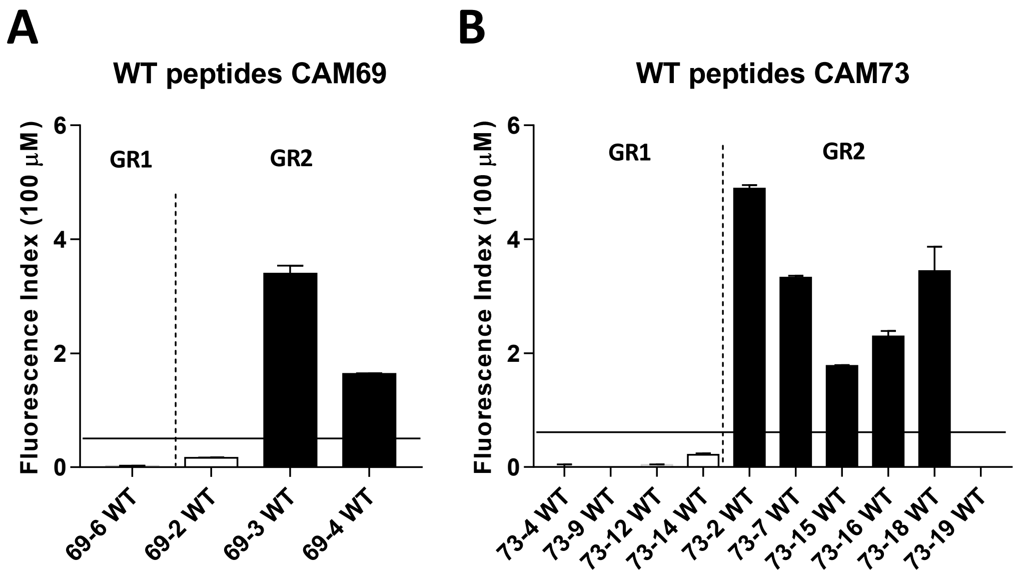

Supplementary Figure S1. Binding to HLA-A2.1 of WT versions of peptides recognized by lymphocytes immunized with mutated epitopes. Peptides from patients CAM69 (A) and CAM73 (B) and recognized by lymphocytes immunized with mutated epitopes were tested at 100 μM in binding assays using T2 cells. Results are expressed as Fluorescence Index and correspond to the mean of duplicate samples in two experiments. Positive binding was considered when FI > 0.5.
